# An Unusual Cause of Vertebral Artery Dissection: Esophagogastroduodenoscopy

**DOI:** 10.4061/2010/915484

**Published:** 2010-08-08

**Authors:** Fernando D. Testai, Philip B. Gorelick

**Affiliations:** ^1^Section of Cerebrovascular Disease and Neurological Critical Care, Department of Neurology and Rehabilitation, College of Medicine at Chicago, University of Illinois, 912 S. Wood Street Room 855N, Chicago, IL 60612, USA; ^2^Section of Cerebrovascular Disease and Neurological Critical Care, Department of Neurology and Rehabilitation, Center for Stroke Research, College of Medicine at Chicago, University of Illinois, 912 S. Wood Street Room 855N, Chicago, IL 60612, USA

## Abstract

Brain-supplying arterial dissection is considered one of the most common vascular causes of stroke in younger patients. Dissections are usually preceded by trauma or mechanical stress; the vascular stressor may be trivial as this condition has been described in association with manipulation and stretching the neck. Here we describe a case of vertebral artery dissection and stroke following esophagogastroduodenoscopy. This case highlights a potentially serious complication that may occur after procedures that require hyperextension of the neck.

## 1. Case

A 74-year-old man with a past medical history of hypertension, coronary artery disease, and Barrett's esophagus presents for evaluation of gait difficulty. The day before the onset of symptoms he had a flexible fiberoptic esophagogastroduodenoscopy (EGD) under general anesthesia with intubation for symptoms of dyspepsia. After recovering from the procedure, the patient noted lack of coordination of the right hand and veering to the right when walking. There was no headache, neck pain, or history of recent trauma to the neck or head.

**Figure 1:**
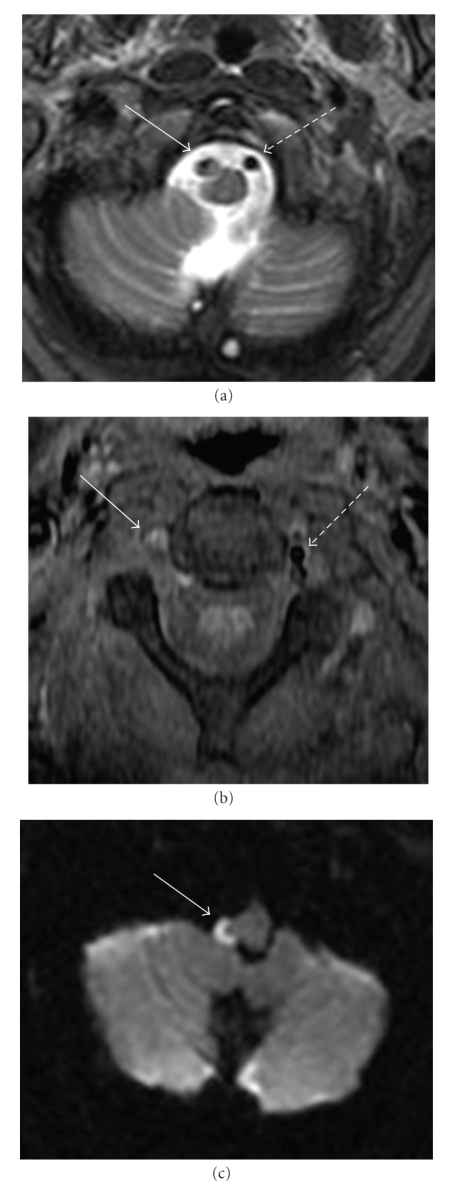
Brain MRI. (a) T2 sequence; a crescent-shaped filling defect is noted in the distal portion of the right VA (solid arrow). This lesion showed enhancement after intravenous infusion with gadolinium. (b) Fat-saturation sequence; a hyperintense lesion is noted in the third segment of the right VA (solid arrow). These findings are consistent with intramural clot formation and suggest vertebral dissection. For comparison purposes, the normal flow void in the left VA is shown (discontinued arrow). (c) Diffusion weighted image; a hyperintense lesion is noted in the right lateral medulla consistent with ischemic stroke.

**Figure 2:**
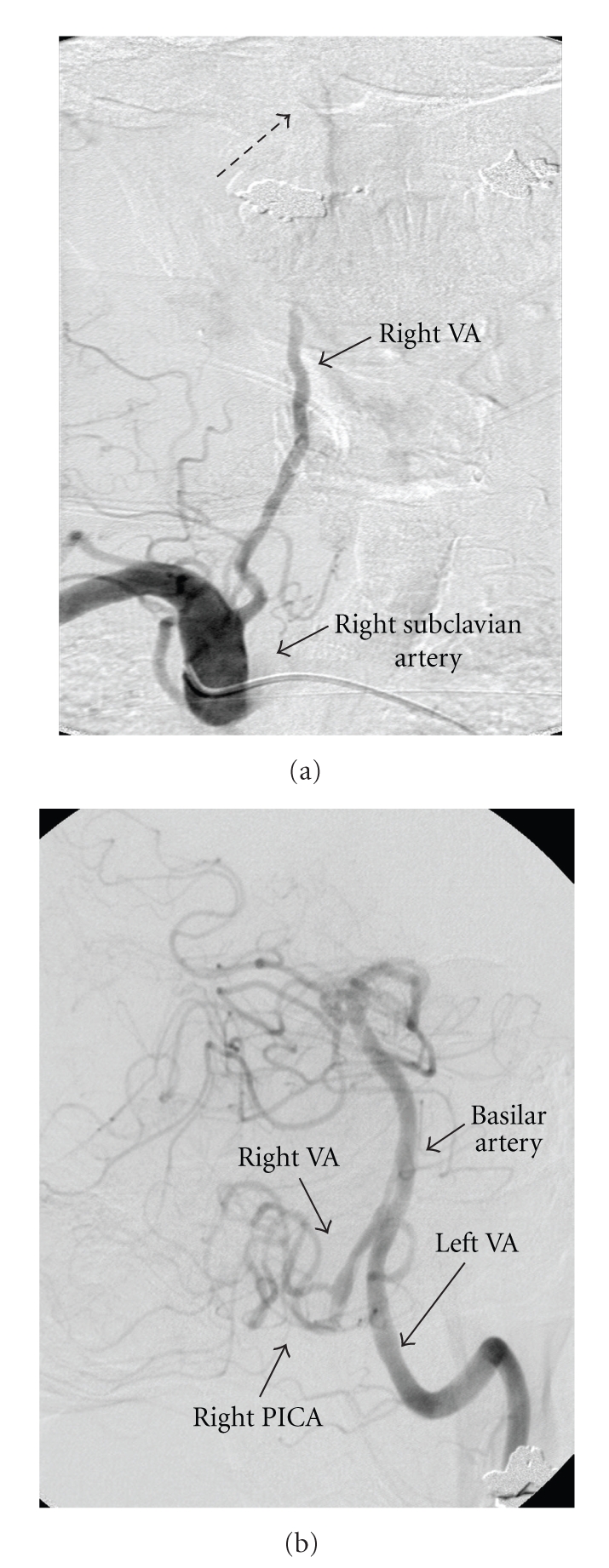
Digital subtraction cerebral angiography. (a) Right subclavian injection. The antegrade contrast flow in the right vertebral artery (VA) terminates just below the level of skull base, at the arch of C1 (discontinuous arrow). (b) Left VA injection. The left VA demonstrates normal course and caliber. There is retrograde contrast filling into the distal portion of the right VA and the right posterior inferior cerebellar artery (PICA). The distal segment of the right VA is irregular suggesting the presence of intraluminal clots.

Neurological examination showed the following signs referable to the right side: partial ptosis, a small poorly reactive pupil to light, decreased vibration and temperature sensation of the arm and leg, finger-to-nose dysmetria, and wide-based gait with veering. On brain MRI, there was an infarct of the right lateral medulla and findings consistent with vertebral artery (VA) dissection (Figure 1). Conventional cerebral angiography showed that the right VA tapered distally in its 3rd segment terminating just below the level of the skull base. The 4th segment of the VA filled retrogradely from the contralateral VA and had an irregular appearance suggestive of thrombus (Figure 2). A diagnosis of right VA dissection was made.

## 2. Discussion

After atherosclerosis, dissection is a common cause of symptomatic in situ carotid artery occlusive disease [1]. Dissections may be classified as traumatic or spontaneous; however, it is increasingly recognized that they may be preceded by trivial mechanical stress as may occur during spinal manipulative therapy [2]. Mechanistically, arterial dissection may begin with a tear in the media resulting in a mural hematoma which may extend proximally and distally. If the intima is damaged, intramurally formed thrombus may enter into the lumen of the vessel and embolize distally. Expansion of the intramural hematoma may lead to compression of the lumen of the vessel creating a low-flow state and distal hypoperfusion. Dissections frequently occur at points where arteries are mobile and not anchored. A common location is above the internal carotid artery (ICA) bifurcation (pharyngeal portion). Also, dissections may occur in the 3rd portion of the VA as it enters the foramen magnum. Fat saturation MRI, CT angiography, MR angiography, and ultrasound examinations are useful for diagnosis, but some consider conventional catheter angiography as the gold standard test [1]. Management of this condition is controversial. Due to the formation of intramural thrombus and the potential for distal embolism, patients are commonly treated in the acute phase with anticoagulants. However, the benefit of this approach over antiplatelet agents has not been systematically studied, and clinical trials to address this issue are needed [3].

Our patient had a partial lateral medullary syndrome due to brain ischemia caused by a right VA dissection in the absence of unintended head maneuvers. In this setting, we hypothesize that the EGD procedure or endotracheal intubation may have led to dissection of the right VA. Hyperextension of the neck, as may occur during intubation, EGD, difficult labor and delivery, or other medical procedures, may lead to stretching and injury of cervical vessels and cause dissection of brain-supplying arteries [4–6]. Alternatively, cerebral hemodynamic changes during sustained hyperextension of the neck have been previously noted in patients with hypoplasia of neck vessels, carotid, and vertebral occlusion, stenosis, or prior ischemic disease, and neck positioning during or after surgery has been proposed to play role in postoperative cerebral ischemia by promoting thrombi formation in compressed arteries that may dislodge and embolize distally when the neck becomes free [7, 8]. Clinicians must think of these diagnostic possibilities when stroke occurs after a medical procedure accompanied by hyperextension of the neck.

## References

[1] Caplan LR (2008). Dissections of brain-supplying arteries. *Nature Clinical Practice Neurology*.

[2] Smith WS, Johnston SC, Skalabrin EJ (2003). Spinal manipulative therapy is an independent risk factor for vertebral artery dissection. *Neurology*.

[3] Georgiadis D, Arnold M, von Buedingen HC (2009). Aspirin vs anticoagulation in carotid artery dissection: a study of 298 patients. *Neurology*.

[4] Riccheüi A, Becher M, Dulguerov P (1999). Internal carotid artery dissection following rigid esophagoscopy. *Archives of Otolaryngology—Head and Neck Surgery*.

[5] Zotter H, Zenz W, Gallistl S, Zöhrer B, Lindbichler F (2000). Stroke following appendectomy under general anesthesia in a patient with basilar impression. *Acta Anaesthesiologica Scandinavica*.

[6] Ringrose T, Thompson W (1999). An unusual case of post operative nausea, vomiting and neck pain. *Critical Care and Resuscitation*.

[7] Weintraub MI, Khoury A (1998). Cerebral hemodynamic changes induced by simulated tracheal intubation: a possible role in perioperative stroke? Magnetic resonance angiography and flow analysis in 160 cases. *Stroke*.

[8] Tettenborn B, Caplan LR, Sloan MA (1993). Postoperative brainstem and cerebellar infarcts. *Neurology*.

